# Noninvasive prenatal diagnosis of 21-Hydroxylase deficiency using target capture sequencing of maternal plasma DNA

**DOI:** 10.1038/s41598-017-06828-2

**Published:** 2017-08-07

**Authors:** Dingyuan Ma, Yuan Yuan, Chunyu Luo, Yaoshen Wang, Tao Jiang, Fengyu Guo, Jingjing Zhang, Chao Chen, Yun Sun, Jian Cheng, Ping Hu, Jian Wang, Huanming Yang, Xin Yi, Wei Wang, Asan, Zhengfeng Xu

**Affiliations:** 1 State key Laboratory of Reproductive Medicine, Department of Prenatal Diagnosis, Nanjing Maternity and Child Health Care Hospital, Obstetrics and Gynecology Hospital Affiliated to Nanjing Medical University, Nanjing, China; 2 Binhai Genomics Institute, BGI-Tianjin, Tianjin, China; 3 Tianjin Translational Genomics Centre, BGI-Tianjin, Tianjin, China; 4 BGI-Shenzhen, Shenzhen, China; 5 James D. Watson Institute of Genome Sciences, Hangzhou, China

## Abstract

Here, we aimed to validate a noninvasive method using capture sequencing for prenatal diagnosis of congenital adrenal hyperplasia due to 21-Hydroxylase deficiency (21-OHD). Noninvasive prenatal diagnosis (NIPD) of 21-OHD was based on 14 plasma samples collected from 12 families, including four plasma sample collected during the first trimester. Targeted capture sequencing was performed using genomic DNA from the parents and child trios to determine the pathogenic and wild-type alleles associated with the haplotypes. Maternal plasma DNA was also sequenced to determine the fetal inheritance of the allele using hidden Markov model-based haplotype linkage analysis. The effect of fetal DNA fraction and sequencing depth on the accuracy of NIPD was investigated. The lower limit of fetal DNA fraction was 2% and the threshold mean sequence depth was 38, suggesting potential advantage if used in early gestation. The *CYP21A2* genotype of the fetus was accurately determined in all the 14 plasma samples as early as day 1 and 8 weeks of gestation. Results suggest the accuracy and feasibility of NIPD of 21-OHD using a small target capture region with a low threshold for fetal DNA fraction and sequence depth. Our method is cost-effective and suggests diagnostic applications in clinical practice.

## Introduction

Congenital adrenal hyperplasia (CAH) is caused by the deficiency of enzymes associated with cortisol synthesis. CAH is a hereditary and an autosomal recessive disease with an incidence of 1 in 10,000 to 1 in 15,000 at birth^1^. Approximately, 90% to 95% of CAH is caused by *CYP21A2*-related 21-hydroxylase deficiency (21-OHD), which leads to increase in adrenal androgen levels. The affected female is virilized or manifests sex reversal, and the affected male displays sexual precocity. Based on the differences in clinical phenotype, this disorder is classified into three types: simple virilizing (SV), salt wasting (SW), and non-classical CAH. The salt wasting type is most severe, and is associated with hyponatremia, hyperkalemia, acidosis, and hypoglycemia. Death due to circulatory failure may occur in the absence of prompt medical intervention^2^.

Prenatal diagnosis facilitates detection of affected fetuses in high-risk families for appropriate medical management. Dexamethasone is indicated in affected females to minimize genital virilization^3^. Prompt medical attention of the affected newborns is essential. However, prenatal diagnosis performed via invasive methods, such as chorionic villus sampling (CVS) and amniocentesis, increase the risk of miscarriage or infection. Recently, many noninvasive prenatal diagnostic methods have been developed to detect fetal genetic status using cell-free fetal DNA (cff-DNA) in maternal plasma^4–7^, for e.g., noninvasive prenatal detection of fetal aneuploidy^8–10^, sex-linked genetic diseases^11, 12^, and autosomal recessive diseases^13–16^. We have successfully reported noninvasive prenatal diagnosis (NIPD) of fetal *CYP21A2* genotype using a combination of target capture sequencing and haplotype-assisted analysis^17^. However, the target region in our previous study spanned the whole exome, and 1 million tag single nucleotide polymorphism (SNP) were captured for sequencing analysis, which may be expensive and low sensitivity for clinical application. New *et al*. reported that target capture sequencing of a 6 Mb region flanking *CYP21A2*, facilitated noninvasive genotype analysis of fetus in CAH using relative haplotype dosage (RHDO) analysis^18^. These studies demonstrated that target capture sequencing can be used in noninvasive prenatal diagnosis of fetal CAH. However, the large targeted region increases sequencing costs, which may limit its routine clinical application.

In this study, we showed that noninvasive prenatal testing (NIPT) of fetal 21-OHD can be accomplished by analyzing a 274.15 kb targeted region, including *CYP21A2* and 1607 surrounding highly heterozygous SNPs distributed within 2 Mbp chromosome 6 region. Inclusion of the chromosome Y-specific sequences in the target region facilitates the simultaneous determination of fetal gender. Our approach suggests an affordable strategy for clinical application of NIPT of monogenic diseases such as 21-OHD.

## Results

### Baseline data of recruited families

The clinical demographics of the studied families are shown in Table 1. The types of pathogenic mutations identified in the 12 families included point mutations, large-scale gene deletions and conversions. Plasma samples were collected twice in the two families, and only once in the other 10 families. The plasma samples in family F-02 were collected in two pregnancies, respectively. In the family F-04, the plasma samples were collected during the first and second trimesters in one pregnancy, respectively. Four out of the 14 plasma samples were collected during the first trimester (ranging from 8 weeks and 1 day to 12 weeks and 2 days), 10 were collected during the second trimester (ranging from 16 weeks and 4 days to 19 weeks and 2 days). All the parents were carriers, 11 children used for haplotype constuction were patients, and one child was normal. The amniocentesis was performed in all the fetuses to sample fetal genomic DNA (gDNA) for invasive molecular diagnosis at 18 weeks and 1 day to 22 weeks and 2 days.

**Table 1:** Overview of the studied families.

Family No.	Pathogenic mutation in *CYP21A2* gene			Plasma sample	Plasma sample collection	Amniotic fluid collection
	Father	Mother	Child			
F-01	Del/WT	p.Arg356Trp/WT	p.Arg356Trp/Del	F-01 plasma	19 wk + 2 d	19 wk + 2 d
F-02	Del/WT	IVS2-13A/C > G/WT	IVS2-13A/C > G/Del	F-02-F plasma (first pregnancy)	19 wk + 1 d	19 wk + 1 d
				F-02-S plasma (second pregnancy)	18 wk + 5 d	18 wk + 5 d
F-03	IVS2-13A/C > G/WT	p.Ile172Asn/WT	IVS2-13A/C > G/p. Ile172Asn	F-03 plasma	16 wk + 4 d	19 wk + 5 d
F-04	p.Ile172Asn/WT	Chimera CH-2/WT	p.Ile172Asn/Chimera CH-2	F-04-8 wk plasma (first trimester)	8 wk + 1 d	
				F-04-18 wk plasma (second trimester)	18 wk + 3 d	18 wk + 3 d
F-05	IVS2-13A/C > G + promoter conversion	Del/WT	IVS2-13A/C > G + promoter conversion/Del	F-05 plasma	16 wk + 6 d	18 wk + 2 d
F-06	Del/WT	p.Ile172Asn/WT	p.Ile172Asn/Del	F-06 plasma	19w	20 wk + 6 d
F-07	Del/WT	IVS2-13A/C > G/WT	IVS2-13A/C > G/Del	F-07 plasma	10 wk + 3 d	20 wk + 1 d
F-08	IVS2-13A/C > G/WT	p.Ile172Asn/WT	IVS2-13A/C > G/p. Ile172Asn	F-08 plasma	10 wk + 4 d	19 wk + 4 d
F-09	p. [Ile172Asn; Arg356Trp]/WT	IVS2-13A/C > G/WT	IVS2-13A/C > G/p. [Ile172Asn; Arg356Trp]	F-09 plasma	12 wk + 2 d	22 wk + 2 d
F-10	p.Arg484ProfsX40/WT	Del/WT	p.Arg484ProfsX40/Del	F-10 plasma	18 wk + 1 d	18 wk + 1 d
F-11^a^	p.[Gln318X; Arg356Trp]/WT	Chimera/WT	WT/WT^a^	F-11 plasma	18 wk + 5 d	18 wk + 5 d
F-12	Partial conv/WT	IVS2-13A/C > G/WT	IVS2-13A/C > G/Partial conv	F-12 plasma	18 wk + 6 d	19 wk + 5 d

^a^The parents were referred to our clinic for prenatal diagnosis because their first two children died shortly after birth due to CAH. Abbreviantions: WT: wild-type allele; Chimera: CH-2, which possesses the *CYP21A1P* gene from exon 1 to exon 4; Partial conv: Cluster E6, p.Val281Leu, p.Leu307PhefsX6, and p.Arg356Trp mutations.

### Effect of fetal DNA fraction and sequencing depth on the accuracy of NIPT

**Figure 1:**
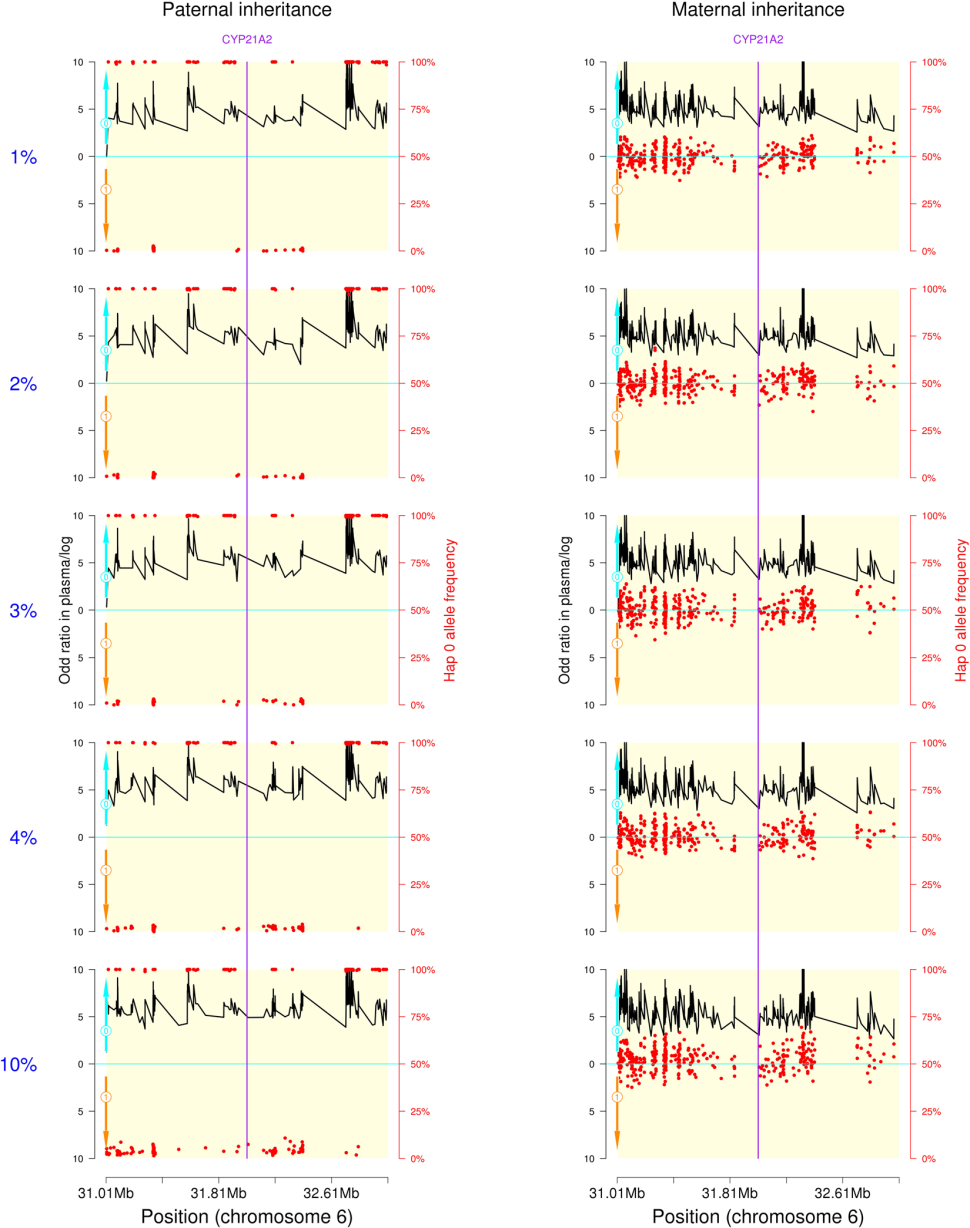
Fetal genotype determination in artificial mixtures. The inheritance of paternal haplotype is displayed on the left, and inheritance of maternal haplotpe on the right. Hap 0 was linked to the pathogenic mutation in *CYP21A2*, while Hap 1 was linked to wild-type allele. The red points represent the frequency of Hap 0 alleles at each informative SNP site. The black lines indicate haplotype inheritance by the fetus. The logarithmic odds ratio of transmission probability between Hap 0 and Hap 1 was calculated by P0*P0re/(P1*P1re), where P0re is the probability of recombination during fetal inheritance Hap 0, and P1re is the probability of recombination if fetus inherited Hap 1.

To investigate the effect of fetal DNA fraction and sequencing depth on the accuracy of our method, we prepared a series of artificial mixtures by mixing the maternal gDNA from family F-02’s and paired fetal gDNA derived from the first pregnancy. Capture sequencing was performed on the samples of parental gDNA, their first child’s gDNA, the fetal gDNA and the artificial mixtures. An average sequencing depth of 268.91 was obtained in these samples (range, 166.63–406.58), with a mean of approximately 99.5% region covered by at least 20 reads. An average of 1.02 million reads (range, 0.79–1.27 million) was mapped to the target region for the artificial samples (Supplemental Table S2). The estimated fetal DNA fractions were 0.94%, 2.16%, 3.15%, 4.08%, and 9.08%, respectively, corresponding to the expected fetal DNA fraction. Using the allele distribution data at each informative SNP site and the SNP linkage relationship obtained from parental haplotypes, a hidden Markov model (HMM) was constructed to calculate the logarithmic odds ratio of transmission probability between the mutant Hap 0 and the wild-type Hap 1 for determined which haplotype is overrepresented in each artificial sample. In the process of decoding the HMM chain, the Viterbi algorithm was used to search for the most probable sequence of hidden states. Haplotype analysis revealed that 830 SNPs were informative for paternal haplotype deduction and 534 SNPs were informative for maternal haplotype deduction in this family. The inferred fetal haplotypes from the HMM are shown in Fig. 1. For paternal haplotype inheritance, the logarithmic odds ratio curves are similar at different fetal DNA fractions. It is the same with maternal haplotype inheritance. An odd ratio above the *x* axe is classified as an allele belong to the mutant Hap 0, whereas an odd ratio below the *x* axe is classified as an allele belong to the wild-type Hap 1. In 2 Mbp genomic region around the *CYP21A2* gene, the fetus inherited the mutant Hap 0 from both parents respectively. These results demonstrated that this approach is robust to the fetal DNA fraction. The scatterplot of the frequency of Hap 0 alleles of the fetus is also shown in Fig. 1. For paternal inheritance, the frequency of the paternal allele (the father is heterozygous) is around half of fetal DNA fraction if it is different from the maternal allele (the mother is homozygous). Conversely, if the paternal allele is the same as the maternal allele, it’s frequency is 100%. For maternal inheritance, the frequency of the maternal allele (the mother is heterozygous) is around 50% whether or not it is the same as the paternal allele (the father was homozygous) because fetal DNA represents only a small fraction of the total maternal plasma DNA. The scatterplot couldn’t give us reliable information about the fetal haplotypes because of site-to-site variability.

The effect of sequencing depth was studied by randomly sampling different numbers of mapped reads for each artificial sample. The statistical and estimated fetal DNA fractions for each artificial data are shown in Supplemental Table S3. A series of simulation data with an average depth of 38.09, 60.25, 110.06, 164.91 and 219.99 were obtained for each artificial mixture with different fetal DNA fraction. For 30× and 50× simulation of 1% fetal DNA fraction artificial mixture, the fetal DNA fractions were too low (0% and 0.5%) to perform haplotype analysis. The results showed that for simulation samples with fetal DNA fraction of 1%, the maternal haplotype could not be correctly determined at any simulation depth. When the fetal DNA fraction reached 2%, a mean sequence depth higher than 38 was adequate to correctly deduce both the paternal and maternal haplotype inheritance (Table 2 and Supplemental Figures S1–S5).

**Table 2:** Effect of fetal DNA fraction and sequencing depth on the accuracy of the fetal haplotype inference in artificial mixtures.

Haplotype	Expected depth	Accuracy of fetal haplotype inference				
		1% fetal DNA fraction	2% fetal DNA fraction	3% fetal DNA fraction	4% fetal DNA fraction	10% fetal DNA fraction
Maternal haplotype	30x	—	100.00%	100.00%	100.00%	100.00%
	50x	—	100.00%	100.00%	100.00%	100.00%
	100x	0	100.00%	100.00%	100.00%	100.00%
	150x	0	100.00%	100.00%	100.00%	100.00%
	200x	0	100.00%	100.00%	100.00%	100.00%
Paternal haplotype	30x	—	100.00%	100.00%	100.00%	100.00%
	50x	—	100.00%	100.00%	100.00%	100.00%
	100x	100.00%	100.00%	100.00%	100.00%	100.00%
	150x	100.00%	100.00%	100.00%	100.00%	100.00%
	200x	100.00%	100.00%	100.00%	100.00%	100.00%

### Plasma sequencing data

The baseline data of each plasma sample are shown in Supplemental Table S4. For genomic DNA samples, the mean depth of region of interest was approximately 183 (range, 73–307) after filtering the duplicate reads, with approximately 96% covered by at least 20 reads. For plasma DNA samples, an average of 1.68 million reads (range, 0.47–2.63 million) was mapped to the target region, the mean depth of region of interest was approximately 223 (range, 91–339) after filtration of the duplicate paired-end reads, with approximately 99.38% region covered by at least 20 reads.

### Estimation of fetal DNA fraction

The fetal DNA fraction in plasma ranged from 5.5% to 15.54% (Table 3). No significant differences were found between the first trimester plasma samples (mean, 8.74%; range, 5.84–12.00%) and the second trimester plasma samples (mean, 10.11%; range, 5.50–15.54%). In the family 4, the fetal DNA fraction in the first trimester (8 wk + 1 d) sample was 7.82%, which increased to 8.15%, in the second trimester sample (18 wk + 3 d). The high fetal DNA fraction observed in the first trimester plasma sample may be attributed to individual difference.

**Table 3:** Determination of fetal sex, fetal DNA fraction, and fetal haplotype in each family.

Plasma sample	Fetal sex	Fetal DNA fraction	Paternal informative SNP	Maternal informative SNP	P-Hap	M-Hap	Fetal genotype	Fetal status
F-01	F	11.08%	769	866	F0	M1	Del/WT	carrier
F-02-F	F	6.88%	830	534	P0	M0	IVS2-13A/C > G/Del	affected
F-02-S	F	9.49%	831	534	P0	M1	Del/WT	carrier
F-03	F	5.50%	675	818	P0	M1	IVS2-13A/C > G/WT	carrier
F-04 8wk	F	7.82%	648	1330	P0	M0	p.Ile172Asn/Chimera	affected
F-04 18wk	F	8.15%	649	1330	P0	M0	p.Ile172Asn/Chimera	affected
F-05	M	8.41%	545	620	P0	M0	IVS2-13A/C > G + promoter conversion/Del	affected
F-06	M	14.28%	800	1024	P0	M1	Del/WT	carrier
F-07	F	9.28%	477	640	P0	M1	Del/WT	carrier
F-08	F	5.84%	524	841	P1	M0	p.Ile172Asn/WT	carrier
F-09	M	12.00%	583	806	P0	M0	IVS2-13A/C > G/p. [Ile172Asn; Arg356Trp]	affected
F-10	M	13.18%	620	829	P1	M0	Del/WT	carrier
F-11	M	15.54%	814	715	P0	M0	p.[Gln318X; Arg356Trp]/Chimera	affected
F-12	F	8.61%	393	711	P0	M0	IVS2-13A/C > G/Partial conv	affected

Abbreviantions: Paternal informative SNP: SNP that was uniquely identified in one paternal haplotype; Maternal informative SNP: SNP that was uniquely identified in one maternal haplotype; Pat-Hap, paternal inherited haplotype; Mat-Hap, maternal inherited haplotype; P0, paternal haplotype linked with pathogenic mutation; P1, paternal haplotype linked with wild-type allele; M0, maternal haplotype linked with pathogenic mutation; M1, maternal haplotype linked with wild-type allele; WT: wild-type allele.

### Noninvasive determination of fetal sex

The fetal sex was determined using the mean depth and 4× coverage of target region in chromosome Y in the plasma samples (Supplemental Table S4). In the paternal gDNA samples, the mean depth of target region in chromosome Y ranged from 180× to 497×, and a mean of 99.9% region was covered by at least 4 reads (range, 98.6–100%). In the maternal gDNA samples, the mean depth and 4× coverage of chromosome Y specific region were extremely low, indicating that the fetal sex could be determined using the two parameters. In the families F-05, F-06, F-09, F-10, F-11, the mean depth of target region in chromosome Y was 59.9 (range, 32.19–76.04), with a mean of 99.20% region covered by at least four reads (range, 97.31–100%), indicating a male fetus in the pregnancy. In the other families, the mean depth of target region in chromosome Y ranged from 2.04 to 11.75, and the 4× coverage ranged from 5.57% to 24.22%, indicating that the fetuses were female. These results were confirmed by the sequencing data of fetal genomic DNA.

### Noninvasive prenatal diagnosis of fetal 21-OHD

**Figure 2:**
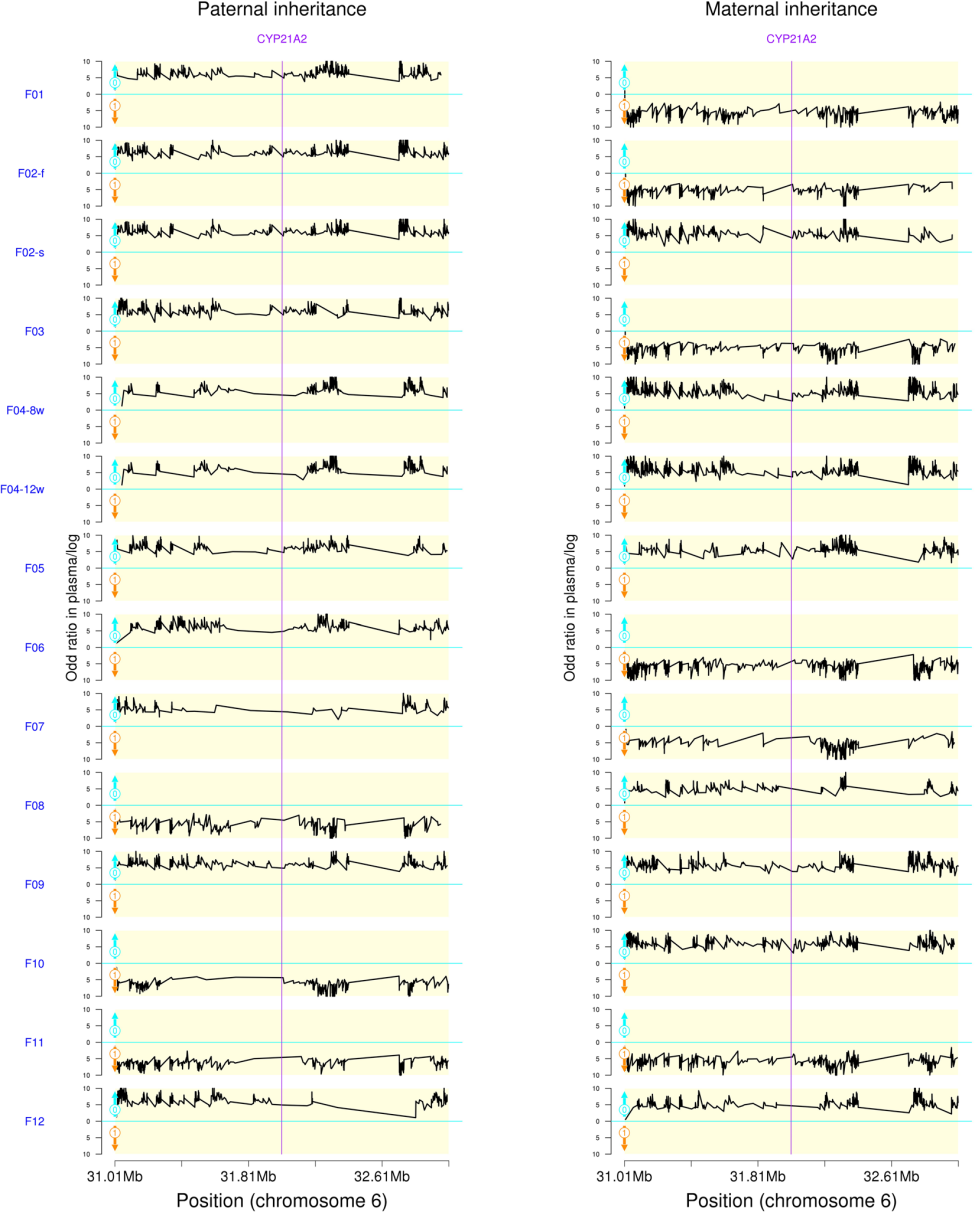
Fetal inheritance of haplotype in families 1 to 12 involving *CYP21A2*. Haplotypes are shown within 2 Mbp genomic region flanking the *CYP21A2* gene. The inheritance of paternal haplotype is illustrated on the left, and the inheritance of maternal haplotpe is shown on the right. Hap 0 is linked to pathogenic mutations in the *CYP21A2*, while Hap 1 is linked to wild-type allele. The black lines indicate haplotype inheritance by the fetus. The logarithmic odds ratio of transmission probability between Hap 0 and Hap 1, and is calculated by P0*P0re/(P1*P1re), where P0re is the probability of recombination involving fetal inheritance of Hap 0, and P1re is the probability of recombination involving fetal inheritance of Hap 1. The purple line indicates the region of *CYP21A2*.

We sequenced 14 plasma DNA samples derived from 12 pregnant women. The sequence data showed high coverage over the region of interest in each sample (Supplemental Table S4). The fetal DNA fraction and sequencing depth of each plasma sample met the requirements for accurate analysis in the simulation samples. An average of 2195 SNPs around the region flanking the *CYP21A2* gene was detected in each of the gDNA samples (Supplemental Table S5). After basic data analysis and SNP calling, parental haplotypes were constructed by trio analysis (analysis of genotypes of the mother, father, and child). The number of informative phased SNPs used for NIPT of 21-OHD ranged from 1104 to 1979 for each family (Table 3). With the assistance of the parental haplotypes, we recovered fetal haplotypes using the HMM using the allele distribution data at informative SNP sites of maternal plasma DNA. The HMM-based prediction of fetal haplotype is shown in Table 3. In order to visualize the prediction results of the HMM, the logarithmic odds ratios of transmission probability between Hap 0 alleles and Hap 1 alleles were calculated at each informative SNP site in 2 Mbp genomic region around the *CYP21A2* gene. These values were then plotted against chromosome position in Fig. 2. Using the first pregnancy sample of family F-02 as an example, involving the genomic region around *CYP21A2*, 1364 SNPs were successfully phased with 830 SNPs to deduce paternal inheritance and with 534 SNPs for maternal inheritance. Mat-Hap 0 was linked to *CYP21A2* deletion, and Pat-Hap 0 was linked to IVS2-13A/C > G mutation. The prediction revealed that the fetus inherited the pathogenic haplotype from both the parents. Thus, the fetal genotype was IVS2-13A/C > G/Del, which was affected by CAH. During the second pregnancy of F-02, the prediction revealed that the fetus inherited the paternal pathogenic haploytpe and the maternal wild-type haplotype, suggesting a carrier with Del/WT genotype. In the 11 remaining samples, six fetuses were predicted to be carriers, and five manifested CAH. In the family 4, similar results were obtained from plasma samples collected at 8 wk + 1 d and 18 wk + 3 d. Amniocentesis was conducted for routine clinical diagnosis of all the fetuses, and the results were consistent with noninvasive diagnosis.

## Discussion

NIPD of monogenic disease is an important clinical goal. It provides requisite prognostic data for clinical intervention. However, in most reported cases, the targeted region for fetal genotype analysis is relatively large, and the cost of sequencing is exorbitant. In this study, we demonstrated the feasibility of NIPD of CAH due to 21-OHD by sequencing a much smaller region. The fetal genotypes were successfully determined from 14 plasma samples obtained from 12 21-OHD families. Based on our previous data^17^, we extensively redesigned and optimized capture probes for targeted sequencing. The target region used for 21-OHD analysis comprised only 274.15 kb, containing the whole *CYP21A2*, highly heterozygous SNPs distributed within 2 Mbp genomic region flanking the *CYP21A2* gene, and the chromosome Y-specific region. Our probe design provided a solution for decreasing the cost of next generation sequencing by increasing the proportion of available data. The high homology between the *CYP21A2* gene and the *CYP21A1P* pseudogene is the major obstacle to a faultless *CYP21A2* genotyping. 21-OHD is genetically caused by a variety of mutations in the *CYP21A2* gene, including point mutations (including small deletions and insertions), large gene deletions (approximately 30 kb) and large gene conversions. Haplotype-based linkage analysis is a promising method to overcome these complications. Our results showed that the developed method enabled us to analyze different types and locations of mutations in the same process.

As an indirect prenatal genetic test, a haplotype-based imbalance analysis for NIPT is limited by the presence of recombination event between target gene and SNP markers. Therefore, there is an indispensable requirement in the test in order to detect recombination events and avoid incorrect prediction. In a recent study, Yoo *et al*. performed a haplotype-based approach for NIPT of DMD after examining and correcting for the recombination event^11^. In one of their families, they identified a recombination event. Although the inherited fetal genotype could be correctly predicted, the approach couldn’t differentiate whether the recombination event occur in the proband or in the new fetus. The authors pointed out that the approach may be biased by false-recombination prediction because informative SNVs might be limited in number and located at greater distance. In our previous study, we constructed the standard haplotype using trio analysis with the SNP genotypes of the parents and amniotic fluid cells^13^. To test the accuracy of the inferred fetal haplotype, we compared the inferred paternal alleles and maternal alleles with the standard haplotypes. In the proband-assisted haplotype phasing (PAHP) process, the result showed that 1,862 paternal alleles and 4,692 maternal alleles were incorrectly phased in terms of a total of 146,103 phased SNP sites. We found 30.12% of the error in the paternal haplotype and 23.66% of the errors in the maternal haplotypes occurred around recombination points. In addition, the PAHP strategy has a lower accuracy as compared with the grandparent-assisted haplotype phasing strategy. How to accurately infer the fetal inherited allele for recombination cases still need the improvement in technology and strategy. To overcome this shortcoming, we restricted the SNP within 2 Mbp genomic region flanking the *CYP21A2* gene to decrease the possibility of the observation of recombination events. The number of informative SNP is another critical factor that influences the accuracy of our method, we captured 1607 highly heterozygous SNPs to guarantee the available of enough informative SNP for each sample (Supplemental Table S5). The small region and high density of highly heterozygous SNPs make the analysis method more accurate and reliable for implementation into clinical practice.

In our study, the parental haplotypes were constructed by using trio analysis based on Mendel’s Law of Segregation. However, the approach is contingent on the availability of DNA from an affected proband and access to specialist facilities with sufficient experience in bioinformatic prediction. These requirements might be difficult to fulfill in certain circumstances. A newly developed technology, microfluidics-based linked-read sequencing, enables the rapid genome wide phasing using a process of microdroplet barcoding of long DNA molecules^19^. Recently, the direct haplotyping technology was first applied in NIPT of single gene diseases^20^. Thirteen families at risk for a fetus with CAH, β-thalassemia, Ellis-van Creveld syndrome, hemophilia or Hunter syndrome were recruited for the study. Utilizing microfluidics-based linked-read sequencing followed by maternal plasma-based RHDO analysis, the mutational status of these 12 fetuses was correctly deduced. The investigators demonstrated that the approach enabled streamline NIPT of single gene diseases without the need to design tailor-made mutation-specific assays and only required the use of specimens from the parents^20^. This represents further progress in the study of NIPD of single gene diseases.

The determination of fetal sex facilitates the prognosis of CAH, and also plays a key role in several monogenic diseases. By adding chromosome Y-specific region to the same capture assay, the fetal sex is determined in a single step. Our results show a significant difference in the coverage and mean depth of chromosome Y between the plasma samples collected from pregnant women with female and male fetuses. Female foetus was associated with very low parameters, which might be caused by non-specific mapping of the reads, to noninvasively distinguish female from male fetuses. The fetal sex was correctly determined in our study.

Expanding the NIPT of 21-OHD to early gestation is meaningful for clinical application. It facilitates comprehensive evaluation by parents and physicians to decide appropriate clinical interventions. In view of the introduction of NIPT-based tests into early gestational application, the fetal DNA fraction is a key parameter governing the analytical performance. In such haplotype-based approaches^11–14, 17, 18^, the influence of fetal DNA on the accuracy of NIPT has not been clarified by using an experimental protocol. We performed a series of simulation experiments to investigate the role of fetal DNA in NIPT of 21-OHD and parameters that might improve the sensitivity of our method. The results showed that at a mean sequence depth of plasma of 39×, the fetal genotype was successfully determined from plasma samples using a fetal DNA fraction of 2%. Most pregnancies show a fetal DNA fraction higher than 4%, suggesting that our method was applicable for use in most pregnancies^21^. We used four early gestation plasma samples (F-02-F, F-07, F-09, and F-10), with a gestation age ranging from 8 wk 1 d to 12 wk 2 d. The fetal DNA fraction of these samples ranged from 5.84% to 13.18%, and the genotype of these four samples was correctly determined. In our study, the fetal DNA fraction of early gestation plasma was not significantly lower than the samples collected during the second trimester, which might be attributed to individual differences and small sample size^21^.

In conclusion, we found that NIPD of 21-OHD based on sequencing of small targeted regions was cost-effective for routine clinical application. We also determined the limitations of fetal DNA fraction and sequencing depth, which indicate that our method favored analysis during early gestation and laid the foundation for further prospective studies.

## Methods and Materials

### Case recruitment

Twelve families diagnosed with CAH were recruited at the Department of Prenatal Diagnosis in Nanjing Maternity and Child Health Care Hospital (Jiangsu, China) between 2014 and 2015. A blood sample was collected from each pregnant woman, her husband, and their first child, and placed into tubes with EDTA to prevent coagulation. Maternal plasma was separated from the blood sample within 8 h. Amniotic fluid samples were obtained through amniocentesis at 18 to 22 weeks of gestation for routine prenatal diagnosis. In plasma DNA analysis, the *CYP21A2* mutations in fetuses were blinded to data analyst engineers. During routine prenatal diagnosis, multiplex ligation-dependent probe amplification (MLPA) and Sanger sequencing were used to identify *CYP21A2* mutations as previously described^22, 23^. Parental informed consent was obtained to perform extensive molecular studies. The present study was conducted in accordance with the Declaration of Helsinki, and was approved by the Ethics Committee of Nanjing Maternity and Child Health Care Hospital (No. [2014] 61).

### Capture sequencing

Cell-free DNA (cf-DNA) was extracted from 1.2 mL samples of maternal plasma using QIAamp Circulating Nucleic Acid Kit (Qiagen, Hilden, Germany), and quantified using Qubit 2.0 fluorometer (Invitrogen by Life Technologies, Carlsbad, CA, USA). A pre-hybridization library was prepared using the KAPA library preparation Kit (KAPA Biosystems, Wilmington, MA, USA). According to the manufacturer’s protocol, 10–20 ng cf-DNA was used for library preparation. After end-repair and “A”-overhanging, adapters were ligated to the DNA fragment, and the library was enriched and labeled with index primers in eight cycles of PCR. The pre-hybridization library of genomic DNA (gDNA) was prepared according to the Illumina standard protocol. Briefly, 1 μg genomic DNA was sonicated to yield DNA fragments with an average size of 200 bp to 250 bp. After end-repairing and “A”-overhanging, the adapters were ligated to the fragments, and the library was enriched and labeled with index primers through 4 to 5 cycles of PCR. The gDNA and plasma DNA libraries were captured using the same custom-designed NimbleGen SeqCap EZ probes (Roche NimbleGen, Madison,WI, USA) with a target region of 274.15 kb, including *CYP21A2*, 1607 highly heterozygous SNPs distributed within 2 Mbp genomic region flanking the *CYP21A2* gene and 13.3 kb chromosome Y-specific region. All the enriched libraries were paired-end sequenced using Illumina Hiseq. 2500 (Illumina, San Diego, CA, USA) with a read length of 101 bp.

### Sequence alignment and SNP calling

The low quality reads were removed using SOAPnuke Software parameter -l 10 -q 0.5 -n 0.1. The BWA software (0.7.12) was used to align reads to the human reference genome (Hg19, GRCh37). The variants in the genomic DNA samples were determined using GATK software, and the low quality reads were filtered out using GATK Variant Filtration (filterExpression “QD < 2.0 || MQ < 40.0 || FS > 60.0 || Haplotype Score > 13.0 || MQ Rank Sum < −12.5 || ReadPos Rank Sum < −8.0”). For plasma DNA sample, a pipeline reported by Garrett M Frampton^24^ was modified to detect variants with allele ratio greater than 1%. Briefly, low quality reads with mapping quality less than 13 or base quality less than 13 were discarded. The PCR duplication reads were marked and removed using Picard (1.87). The variants with an allele ratio greater than 1% were determined by Bayesian methodology, the strand bias was filtered using Fisher’s test (P < 1e-4) and the read location bias was filtered using Kolmogorov-Smirnov test (P < 1e-6). We also filtered out sites covered by less than 40 reads.

### Calculation of fetal DNA fraction

The fetal DNA fraction was calculated using SNPs with different parental homozygosity, using the following formula:

1 $$f=\frac{\sum _{i}^{n}\frac{2{p}_{i}}{({p}_{i}+{q}_{i})}}{n}$$

where *p*_*i*_ denotes the number of paternal specific alleles and *q*_*i*_ is the number of maternal alleles in the plasma sample, and n is the number of SNPs using fetal DNA fraction.

### Determination of the fetal sex

Plasma samples of male fetus showed higher mean depth of target region on chromosome Y and 4× coverage than in female fetus. Thus, the fetal sex was determined according to the mean depth and 4× coverage of the 13 kb chromosome Y-specific region.

### Noninvasive prenatal detection of fetal *CYP21A2* genotype

Base on the trios’ strategy^17, 18^, the parental haplotypes and their linkage to 21-OHD were established using SNPs identified in the *CYP21A2* gene and its flanking region in the parents and the child. We defined haplotype 0 (Hap 0) as the haplotype linked to the pathogenic mutation, and haplotype 1 (Hap 1) as the haplotype linked to the wild-type allele. Paternal inheritance was determined using SNPs heterozygous in father but homozygous in mother. Maternal inheritance was determined using SNPs that were heterozygous in mother but homozygous in father. We defined haplotype-informative SNP as SNP that was uniquely identified in one haplotype, and not in the other three haplotypes. Informative SNPs linked to the inherited haplotype are over-represented in the plasma. We constructed a hidden Markov model (HMM)^17, 25^ to determine the inheritance of parental haplotype from plasma data.

Briefly, for each SNP, the observed state was denoted as S = {N_j_}, where N_j_ = {Pos, Fgt, Mgt, Pgt, dep_ref, dep_alt}, and Pos, Fgt, Mgt, Pgt, dep_ref, dep_alt denote position, paternal genotype, maternal genotype, child’s genotype, the number of reads supporting the reference allele and non-reference allele, respectively.

The hidden state was defined as the pathogenic haplotype (Hap 0) inherited by the fetus, and was denoted as Q = {Hap 0, Hap 1}. Because of the lack of prior probability, we defined the initial state distribution as π = {1/2, 1/2}.

The emission probabilities matrix is represented by B = {b_i,j_}, b_i,j_ = P{Hap i|N_j_}, *i* ∈{0, 1}, j = 1, 2, 3, …, n. The probability of Hap i-rich SNP transmitted to the fetus at a given site j was calculated as follows:

2 $$P\{Hapi|{N}_{j}\}=\frac{P\{{N}_{j}|Hapi\}\times 0.5}{P\{{N}_{j}|Hap\,0\}\times P\{{N}_{j}|Hap\,1\}\times 0.5}$$

P{Nj | Hap 0} and P{Nj | Hap 1} were calculated using binomial distribution:

3 $${\rm{P}}\{{\rm{Nj}}|{\rm{Hap}}\,0\}={\rm{b}}({\rm{k}},{\rm{n}},{\rm{prob}}0)$$

4 $${\rm{P}}\{{\rm{Nj}}|{\rm{Hap}}\,1\}={\rm{b}}(k,n,\mathrm{prob}1)$$

P{Nj| Hap i} is the probability of the observed state Nj at a given SNP loci j in the plasma when Hap i was transmitted to the fetus, k denotes the reads of the haplotype-rich allele, n refers to the total reads at a given site, prob0 and prob1 represent the expected proportion of Hap 0 infomative allele and Hap 1 informative alleles calculated according to fetal DNA fraction, respectively, as illustrated in Supplemental Table S1.

The transition probabilities matrix was denoted as A = {a_j_}, where

5 $${a}_{j}=(\begin{array}{cc}1-{P}_{{\rm{j}}} & {P}_{j}\\ {P}_{j} & 1-{P}_{j}\end{array})$$

and P_j_ is the probability of recombination between two neighboring SNPs calculated by the genetic distance determined using the hapmap.

Finally, the Viterbi algorithm^26^ was used to determine the optimal path through the observed data and deduce the inheritance of maternal haplotype and paternal haplotype, respectively:

6 $$Path[i]={\sum }_{j}^{n}\mathrm{ln}(\mathop{{\rm{\max }}}\limits_{r\in (0,1)}{{\rm{b}}}_{i,j}\times ({a}_{i+1,r+1,j}))$$

### Role of fetal DNA fraction and sequencing depth in NIPT

To determine the effect of fetal DNA fraction and sequencing depth on the accuracy of NIPT, we performed experiments using a series of artificial samples of fetal gDNA diluted to paired maternal gDNA. Briefly, the maternal gDNA and fetal gDNA of case 2′ first pregnancy were fragmented to ~200 bp using sonicator. Double size selection of fragments was performed using SPRIselect beads (Beckman Coulter Genomics, Danvers, MA, USA) to recover fragment size 100–300 bp according to the manufacturer’s protocol. After quantification using Qubit (Invitrogen by Life Technologies), fetal gDNA and maternal gDNA were combined to create artificial plasma samples of 1%, 2%, 3%, 4%, and 10% fetal DNA fractions (w/w). Sequencing libraries were prepared using the same protocol as real plasma sample, and sequenced with pair-ended 101 bp reads using HiSeq. 2500 (Illumina). To investigate the effect of sequencing depth on the accuracy of NIPT, for each artificial plasma sample, we simulated a series of sequencing depths at 30×, 50×, 100×, 150×, and 200× by randomly sampling different numbers of mapped reads. The simulated data were analyzed using our NIPT method. The accuracy of our method was determined by comparing the deduced fetal haplotype of *CYP21A2* and the flanking SNPs with that of the standard haplotype deduced from fetal genomic DNA.

## Electronic supplementary material

Supplementary Information
