# Supplementary material for: Noninvasive prenatal diagnosis of 21-Hydroxylase deficiency using target capture sequencing of maternal plasma DNA
Source: Sci Rep. 2017 Aug 7;7:7427. doi: 10.1038/s41598-017-06828-2 (PMC5547133; doi:10.1038/s41598-017-06828-2)
Supplement: Supplementary file 1 — Supplementary Information [file 41598_2017_6828_MOESM1_ESM.pdf]

Noninvasive prenatal diagnosis of 21-Hydroxylase deficiency using target capture sequencing of maternal plasma DNA

Dingyuan Ma[1,+], Yuan Yuan[2,3,4,+], Chunyu Luo[1,+], Yaoshen Wang[2,3,4], Tao Jiang[1], Fengyu Guo[2,3,4], Jingjing Zhang[1], Chao Chen[2,3,4], Yun Sun[1], Jian Cheng[1], Ping Hu[1], Jian Wang[4,5], Huanming Yang[4,5], Xin Yi[4], Wei Wang[2,3,4], Asan[2,3,4,*] & Zhengfeng Xu[1,*]

[1]State key Laboratory of Reproductive Medicine, Department of Prenatal Diagnosis, Nanjing Maternity and Child Health Care Hospital, Obstetrics and Gynecology Hospital Affiliated to Nanjing Medical University, Nanjing, China; [2]Binhai Genomics Institute, BGI-Tianjin, Tianjin, China; [3]Tianjin Translational Genomics Centre, BGI-Tianjin, Tianjin, China; [4]BGI-Shenzhen, Shenzhen, China; [5]James D. Watson Institute of Genome Sciences, Hangzhou, China.

Supplemental Figure Legends

Figure S1. Determination of fetal haplotype in artificial mixtures containing fetal DNA fractions of 1% and different sequencing depths.

Figure S2. Determination of fetal haplotype in artificial mixtures containing fetal DNA fractions of 2% and different sequencing depths.

Figure S3. Determination of fetal haplotype in artificial mixtures containing fetal DNA fraction of 3% and different sequencing depths.

Figure S4. Determination of fetal haplotype in artificial mixtures containing fetal DNA fractions of 4% and different sequencing depths.

Figure S5. Determination of fetal haplotype in artificial mixtures containing fetal DNA fractions of 10% and different sequencing depths.

Supplemental Table Legends

Table S1. The expected proportion of Hap 0 and Hap 1 informative alleles calculated according to fetal DNA fraction.

Table S2. Sequencing data and estimation of fetal DNA fractions of each artificial mixture.

Table S3. Sequencing data of artificial mixtures under different simulation depths.

Table S4. Sequencing data of genomic DNA and plasma DNA samples.

Table S5. Number of SNPs identified in each family.

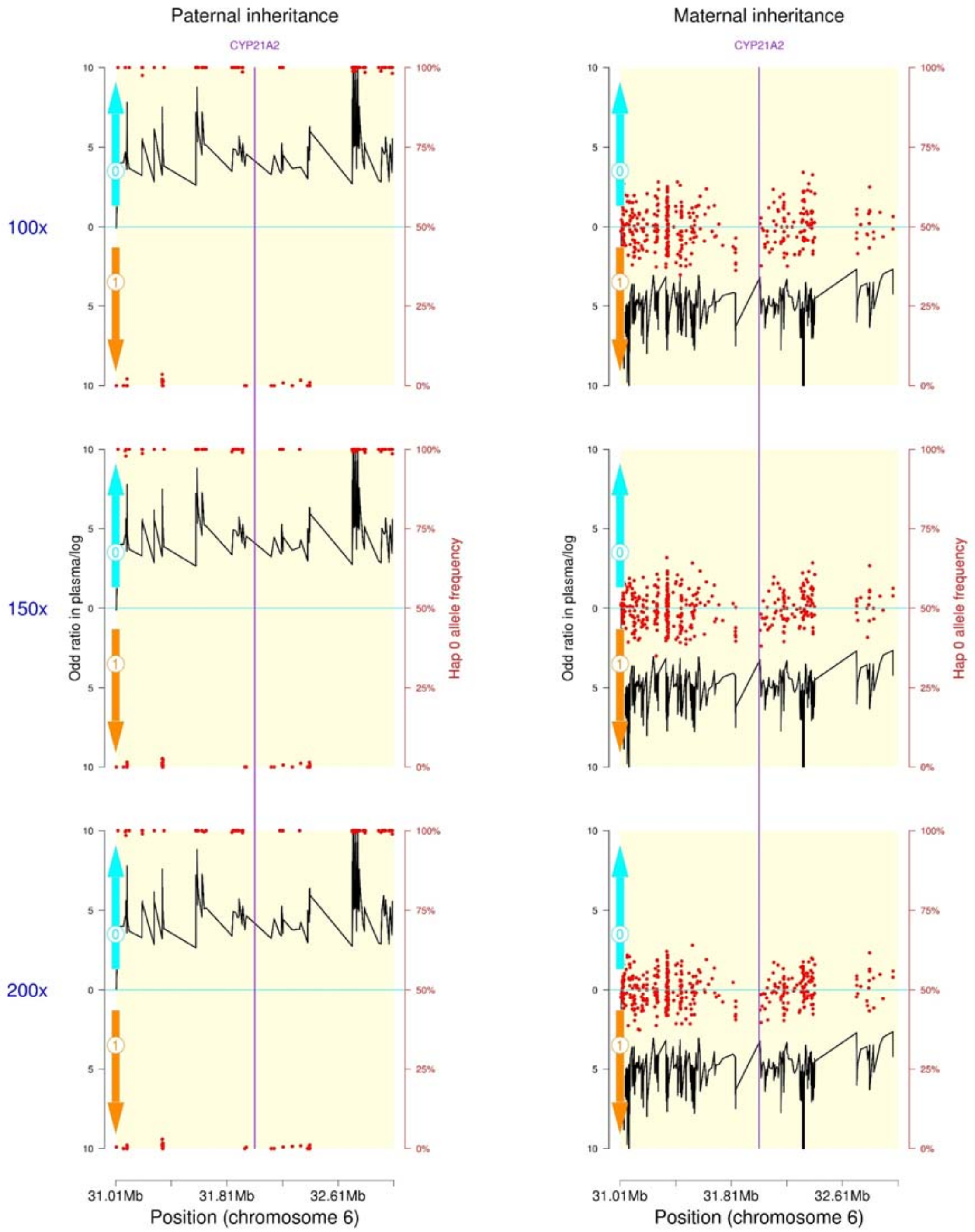

Figure S1. Determination of fetal haplotype in artificial mixtures containing fetal DNA fractions of 1% and different sequencing depths.

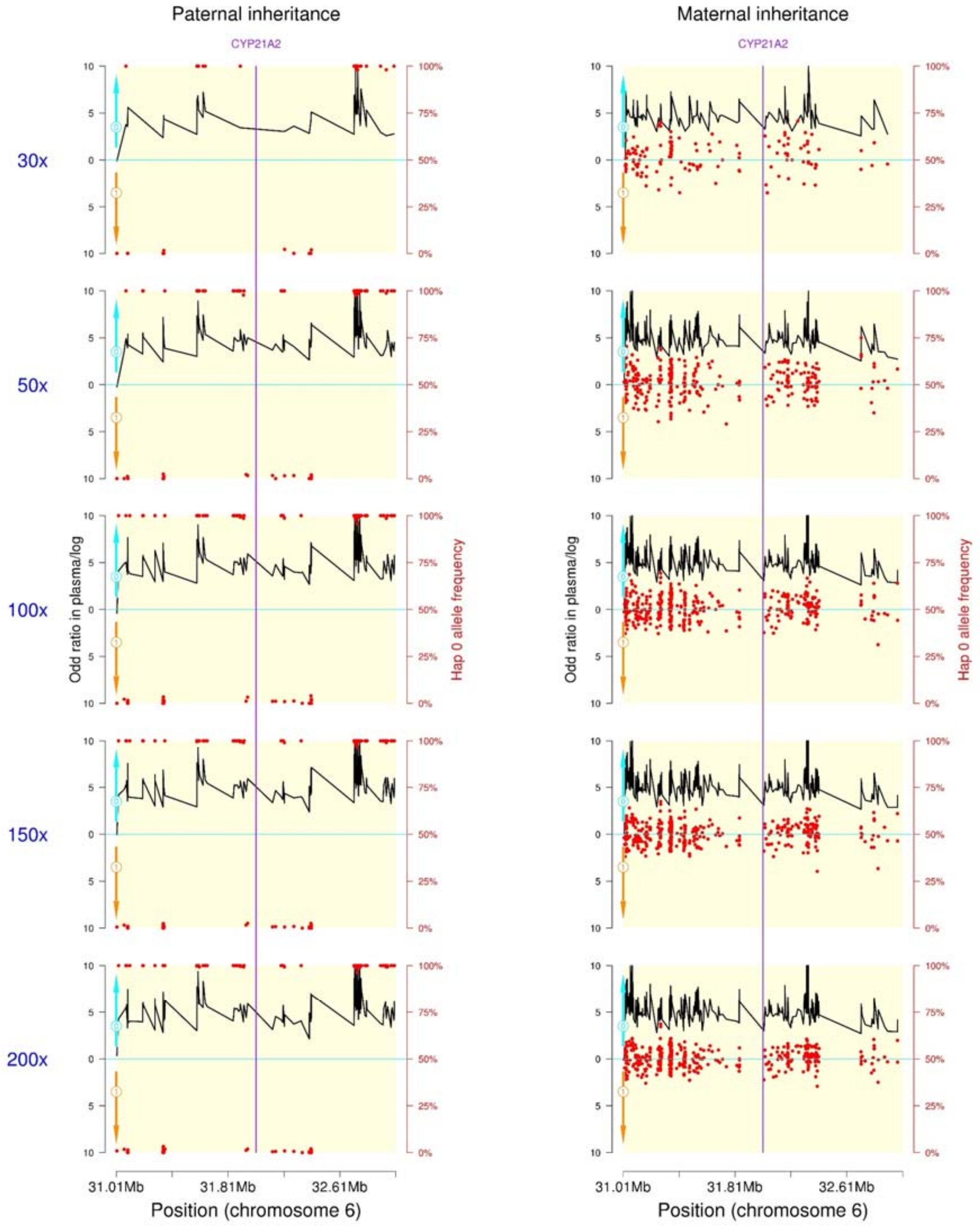

Figure S2. Determination of fetal haplotype in artificial mixtures containing fetal DNA fractions of 2% and different sequencing depths.

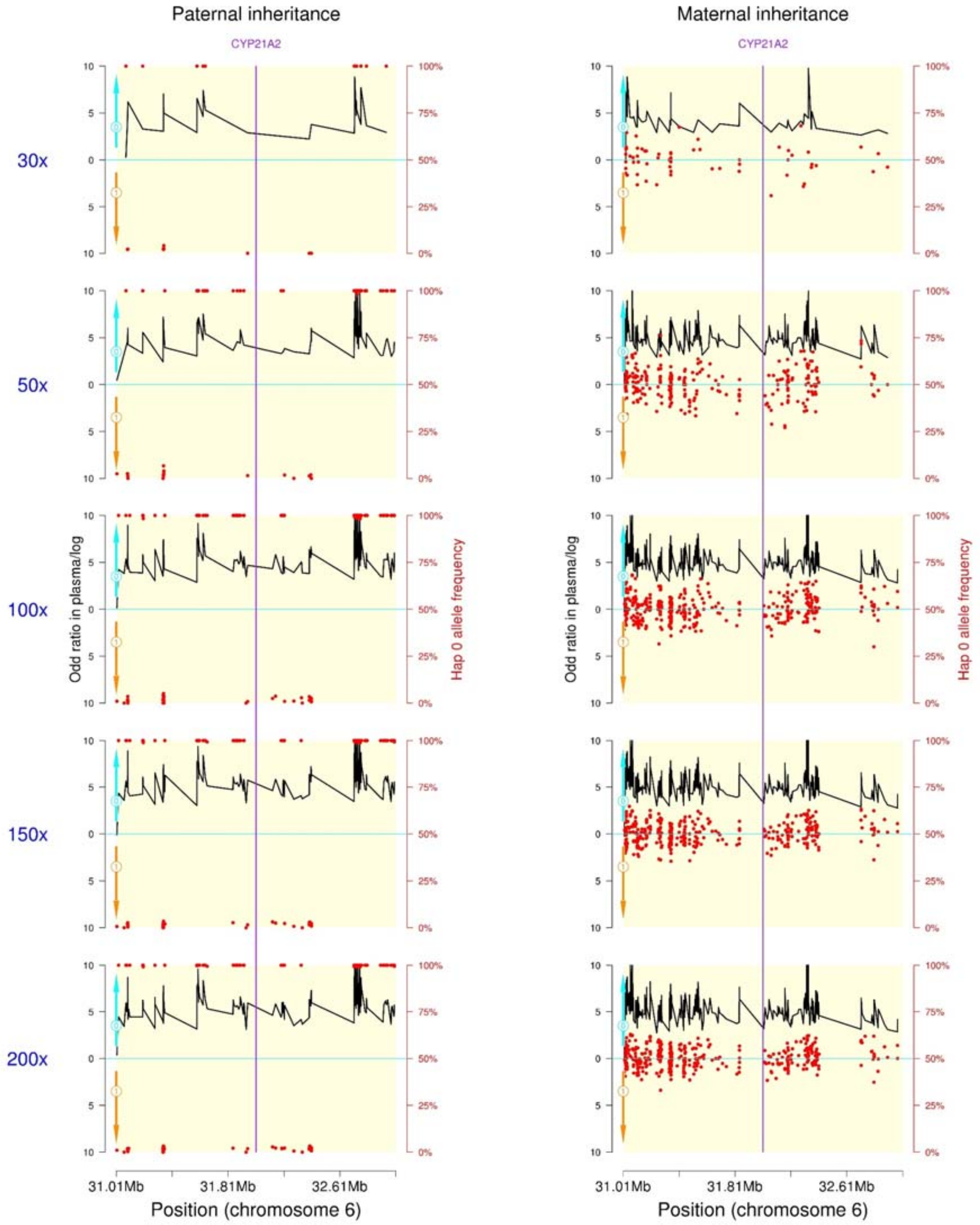

Figure S3. Determination of fetal haplotype in artificial mixtures containing fetal DNA fraction of 3% and different sequencing depths.

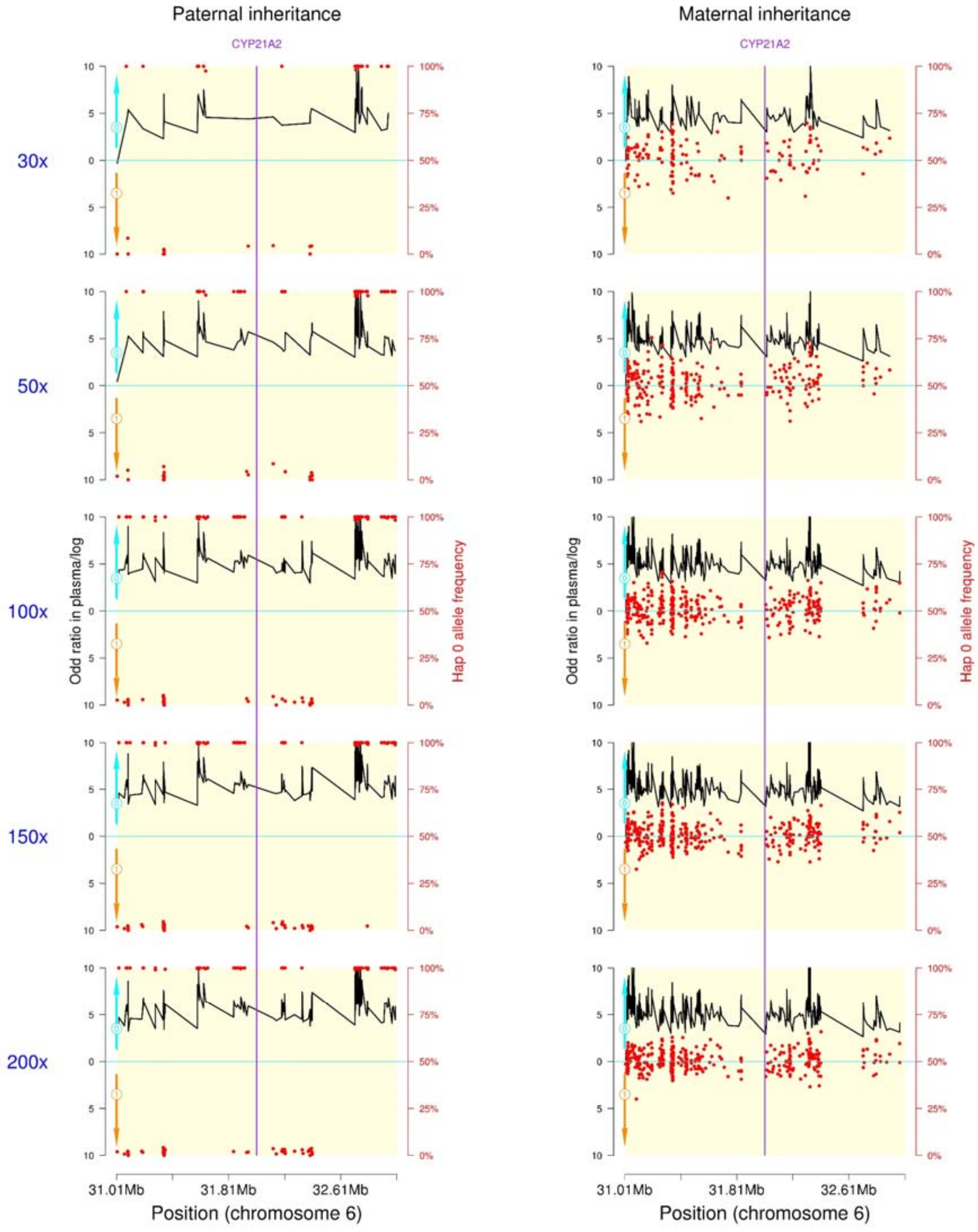

Figure S4. Determination of fetal haplotype in artificial mixtures containing fetal DNA fractions of 4% and different sequencing depths.

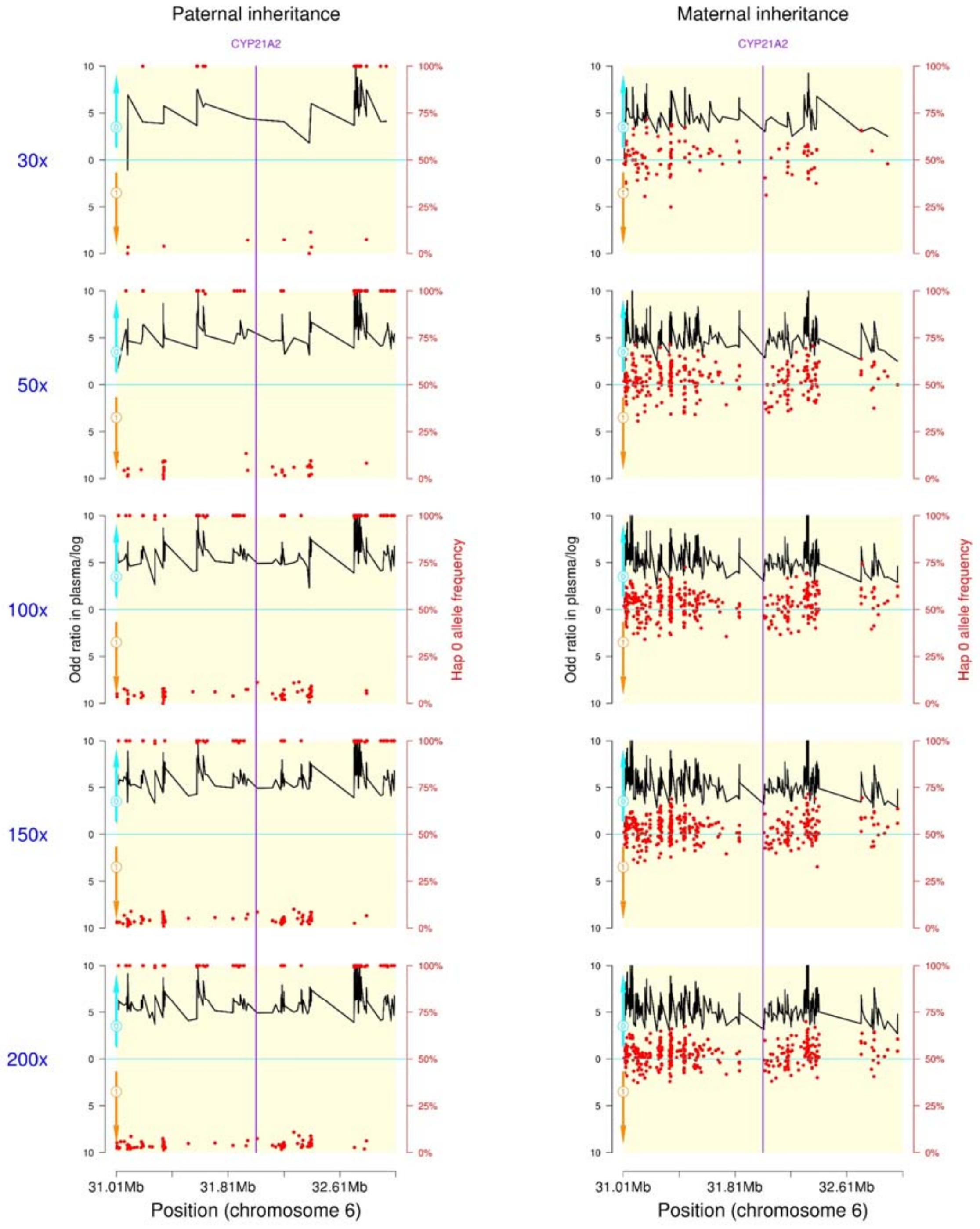

Figure S5. Determination of fetal haplotype in artificial mixtures containing fetal DNA fractions of 10% and different sequencing depths.

Paternal genotype	Maternal genotype	Hap 0 genotype	Expected proportion of Hap 0 informative allele	Expected proportion of Hap 1 informative allele
0/0	0/1	0	(1-f)/2	0.5
0/0	0/1	1	0.5	(1-f)/2
1/1	0/1	0	0.5	(1-f)/2
1/1	0/1	1	(1-f)/2	0.5
0/1	0/0	0	Perr	0.5f
0/1	0/0	1	0.5f	Perr
0/1	1/1	0	0.5f	Perr
0/1	1/1	1	Perr	0.5f

Table S1. The expected proportion of Hap 0 and Hap 1 informative alleles calculated according to fetal DNA fraction. f: fetal DNA fraction; Perr: sequencing error rate.

Sample	Reads mapped to target region (M)	Mean depth of target region*	Fraction of target covered ≥20×	Mean depth of target region in ChrY*	Fraction of target covered ≥4× in ChrY	Estimated fetal DNA fraction
Mother	0.49	150.09	98.14%	0.00	0.00%	-
Father	0.63	190.85	99.50%	251.75	100.00%	-
Child	0.81	171.77	99.26%	5.15	8.27%	-
Amniotic fluid-F	0.57	173.85	99.32%	5.14	5.80%	-
1% fetal DNA fraction	1.21	310.76	99.96%	8.79	5.66%	0.94%
2% fetal DNA fraction	0.97	256.84	99.90%	7.75	5.57%	2.16%
3% fetal DNA fraction	0.86	233.21	99.90%	7.30	5.85%	3.15%
4% fetal DNA fraction	1.27	319.93	99.97%	10.17	5.75%	4.08%
10% fetal DNA fraction	0.79	219.51	99.90%	6.79	6.26%	9.08%

Table S2. Sequencing data and estimation of fetal DNA fractions of each artificial mixture. *

After filtration of the duplicated paired-end reads.

Artificial mixture	Expected mean depth	Reads mapped to target region(M)	Mean depth of target region*	Fraction of target covered≥20×	Estimated fetal DNA fraction
1% fetal DNA fraction	30	0.13	40.32	86.99%	0.00%
	50	0.22	66.24	96.36%	0.58%
	100	0.43	110.42	99.12%	0.92%
	150	0.65	165.40	99.74%	0.89%
	200	0.86	220.54	99.91%	0.88%
2% fetal DNA fraction	30	0.13	39.23	86.12%	1.44%
	50	0.21	64.23	95.87%	1.81%
	100	0.42	111.12	99.10%	1.92%
	150	0.62	166.15	99.72%	2.08%
	200	0.83	221.24	99.88%	2.08%
3% fetal DNA fraction	30	0.12	33.06	80.36%	2.63%
	50	0.20	55.11	94.75%	2.49%
	100	0.41	109.91	99.09%	2.94%
	150	0.61	164.88	99.75%	3.05%
	200	0.82	220.08	99.87%	3.16%
4% fetal DNA fraction	30	0.13	40.95	88.63%	3.47%
	50	0.22	54.98	95.17%	3.82%
	100	0.43	109.80	99.15%	4.02%
	150	0.65	164.68	99.75%	4.30%
	200	0.87	219.92	99.90%	4.26%
10% fetal DNA fraction	30	0.12	36.92	84.53%	9.96%
	50	0.20	60.72	95.76%	11.10%
	100	0.39	109.03	99.10%	10.21%
	150	0.59	163.45	99.72%	9.57%
	200	0.79	218.16	99.90%	9.06%

Table S3. Sequencing data of artificial mixtures under different simulation depths. *After filtration of the duplicated paired-end reads.

Family No.	Samples	Reads mapped to target region (M)	Mean depth of target region*	Fraction of target covered ≥20× (%)	Mean depth in chr Y*	Fraction of target covered ≥4 × in chr Y (%)
F-01	mother	0.55	170.12	99.34%	0.00	0.00%
	father	0.60	182.14	99.24%	250.32	100.00%
	child	0.55	168.92	99.35%	239.01	100.00%
	Amniotic fluid	0.84	181.32	98.79%	3.92	6.15%
	plasma	1.97	265.56	99.88%	3.56	5.57%
F-02	mother	0.49	150.09	98.14%	0.00	0.00%
	father	0.63	190.85	99.50%	251.75	100.00%
	child	0.81	171.77	99.26%	5.15	8.27%
	Amniotic fluid-F	0.57	173.85	99.32%	5.14	5.80%
	Amniotic fluid-S	0.86	185.36	99.35%	4.08	5.55%
	Plasma-F	2.57	339.95	99.65%	11.27	6.86%
	Plasma-S	2.31	293.24	99.36%	11.75	5.98%
F-03	mother	0.53	161.26	98.23%	0.00	0.00%
	father	0.60	180.10	99.49%	236.91	100.00%
	child	0.61	186.46	99.47%	3.54	5.58%
	Amniotic fluid	1.20	244.74	99.64%	7.03	6.58%
	plasma	1.61	257.90	99.02%	9.39	6.35%
F-04	mother	1.63	307.12	99.92%	0.00	0.00%
	father	1.14	225.94	99.51%	435.79	100.00%
	child	1.35	260.22	99.80%	4.76	8.40%
	Amniotic fluid	0.93	196.16	99.71%	6.11	7.53%
	Plasma-8wk	2.48	218.27	99.95%	6.56	5.79%
	Plasma-16wk	1.85	217.11	99.86%	8.80	6.70%
F-05	mother	1.37	262.54	99.59%	0.00	0.00%
	father	1.35	259.90	99.71%	489.64	100.00%
	child	1.11	213.36	99.81%	426.40	100.00%
	Amniotic fluid	1.21	252.50	99.78%	475.46	100.00%
	plasma	2.63	310.95	99.87%	61.05	99.16%
F-06	mother	1.20	230.72	99.90%	0.00	0.00%
	father	1.24	235.52	99.57%	497.34	100.00%
	child	0.85	170.66	99.34%	3.60	5.61%
	Amniotic fluid	0.93	199.04	99.77%	387.37	100.00%
	plasma	1.36	106.86	99.65%	32.19	97.31%
F-07	mother	0.50	86.75	91.06%	0.86	2.98%
	father	0.48	85.43	91.66%	180.72	98.63%

	child	0.42	73.72	89.09%	1.53	4.42%
	Amniotic fluid	0.57	132.55	99.20%	3.05	5.72%
	plasma	0.47	91.31	96.72%	2.04	14.54%
F-08	mother	1.31	195.84	99.55%	3.34	25.57%
	father	1.36	203.35	99.51%	402.18	100.00%
	child	1.49	219.23	99.54%	7.42	42.18%
	Amniotic fluid	1.06	226.33	99.58%	4.71	5.92%
	plasma	0.91	175.16	99.43%	3.55	24.22%
F-09	mother	0.77	145.83	99.20%	0.00	0.00%
	father	0.79	153.84	99.02%	279.23	100.00%
	child	0.66	127.49	98.90%	260.43	100.00%
	Amniotic fluid	1.06	230.56	99.69%	423.01	100.00%
	plasma	1.47	236.83	99.64%	64.74	99.51%
F-10	mother	0.69	135.15	98.91%	0.00	0.00%
	father	0.84	161.58	99.59%	290.36	100.00%
	child	0.51	106.69	98.85%	2.98	5.29%
	Amniotic fluid	1.10	239.69	99.44%	437.83	100.00%
	plasma	1.44	242.52	99.33%	76.40	100.00%
F-11	mother	0.83	158.35	99.00%	0.00	0.00%
	father	0.75	146.66	99.24%	277.47	100.00%
	child	0.95	192.24	99.48%	6.12	5.94%
	Amniotic fluid	0.90	198.13	99.11%	369.44	100.00%
	plasma	1.36	195.46	99.42%	65.21	100.00%
F-12	mother	0.61	122.47	99.14%	0.00	0.00%
	father	0.65	129.24	96.90%	238.51	100.00%
	child	0.72	142.53	99.16%	6.50	5.55%
	Amniotic fluid	1.02	226.38	99.77%	6.93	5.56%
	plasma	1.19	183.14	99.60%	7.13	6.33%

Table S4. Sequencing data of genomic DNA and plasma DNA samples. *After filtration of the duplicated paired-end reads.

Family No.	SNP identified in maternal gDNA	SNP identified in Paternal gDNA	SNP identified in child's gDNA
F-01	1800	1746	1711
F-02-F	1890	2028	1732
F-02-S	1892	2030	1734
F-03	1880	1526	1791
F-04 8wk	2097	1881	1918
F-04 18wk	2101	1887	1922
F-05	2003	1967	1823
F-06	1952	2097	2100
F-07	3924	3619	4064
F-08	4542	4175	4121
F-09	2205	2187	2281
F-10	1821	1605	1699
F-11	1990	1920	1875
F-12	1493	1460	1728

Table S5. Number of SNPs identified in each family.
